# Association Between Consumption of Sugar-Sweetened Beverages and 100% Fruit Juice With Poor Mental Health Among US Adults in 11 US States and the District of Columbia

**DOI:** 10.5888/pcd18.200574

**Published:** 2021-05-20

**Authors:** Sophia L. Freije, Camilla C. Senter, Aspen D. Avery, Stephen E. Hawes, Jessica C. Jones-Smith

**Affiliations:** 1 University of Washington School of Public Health, Seattle, Washington

## Abstract

**Introduction:**

Excess sugar consumption is linked to several mental health conditions. Sugar-sweetened beverages (SSBs) and 100% fruit juice contain similar amounts of sugar per serving, yet prior studies examining sugary beverages and mental health are limited to SSBs. Of those, few have assessed potential modifiers such as sex.

**Methods:**

We examined the association between daily consumption of fruit juice and SSBs with poor mental health by using data from the 2017 Behavioral Risk Factor Surveillance System. We used Poisson regression models with clustered-robust standard errors to measure the association between SSB and fruit juice consumption (none, >0 to <1, and ≥1 times per day) and experiencing 14 or more days of poor mental health in the past month, adjusting for sociodemographic characteristics. We used an F test of joint significance to assess effect modification by sex for SSB and fruit juice analyses.

**Results:**

Consuming SSBs 1 or more times per day versus consuming none was associated with a 26% greater prevalence of poor mental health (95% CI, 1.11–1.43). Associations for consuming >0 to <1 times per day compared with consuming none were not significant. We found no evidence of an association between fruit juice consumption and mental health, nor evidence of effect modification by sex in the SSB and fruit juice analyses.

**Conclusion:**

Consuming SSBs 1 or more times per day was significantly associated with poor mental health whereas 100% fruit juice consumption was not. Future studies should examine alternative cut-points of fruit juice by using prospective designs.

**Summary**

**What is already known on this topic?**

Sugary beverages are a leading dietary source of sugar in the United States, and overconsumption of sugar is a hypothesized contributor to poor mental health.

**What is added by this report?**

We examined the association between daily consumption of fruit juice and sugar-sweetened beverages (SSBs) with poor mental health and found that consuming SSBs 1 or more times per day versus consuming none was associated with poor mental health and that frequency of 100% fruit juice consumption was not associated with poor mental health.

**What are the implications for public health practice?**

To improve understanding of the differential effect of SSBs and 100% fruit juice on mental health, future studies should leverage prospective designs to assess the relationship between specific volumes and subtypes of sugary beverage consumption and poor mental health.

## Introduction

Poor mental health is a major concern in the United States, where almost 50% of people are diagnosed with a mental health condition during their lifetime (1). Good mental health is typically defined as a state of well-being, the ability to cope with life challenges, and the absence of persistent mental distress (2). A healthy diet is a well-documented correlate of good mental health (3), and recent randomized controlled trials have demonstrated that healthy diet modifications precede improvements in mental health (4).

Overconsumption of sugar is a hypothesized contributor to poor mental health through several plausible biological pathways (5,6). Sugary beverages are a leading dietary source of sugar in the United States (7), where a typical serving meets or exceeds the recommended daily intake (8). Previous studies have examined mental health in relation to consumption of sugar-sweetened beverages (SSBs) or other beverages containing added sugar. Two studies linked general SSB consumption with recurrent depression and mental distress (9,10), and 1 meta-analysis reported consuming SSBs versus consuming none was associated with 31% higher odds of depression (11). Because most of these studies were limited to caffeinated soda, little is known about the relationship between other sweetened beverages and mental health.

Beverages containing naturally derived sugar such as 100% fruit juice have not been examined in relation to mental health, although they contain similar total sugar levels and calories per serving to SSBs (12). Although consuming a typical 8- to 12-ounce serving of naturally sweetened beverages is considered healthy (8), consumption of larger quantities of 100% fruit juice has been linked to several adverse health outcomes including type 2 diabetes (13) and all-cause mortality (14).

Sex may modify the relationship between diet and mental health through both biological and behavioral mechanisms, including differences in emotional regulation by gonadal hormones (15) and greater perceived stress among females in response to consuming unhealthy foods (16,17). Furthermore, a typical serving size of the same beverage may affect men and women differently because of differences in body size and composition. A few studies have examined how SSB consumption and mental health differ by sex, but the findings are mixed (18–20).

We examined associations between consumption of SSBs and 100% fruit juice and experiencing 14 or more days of poor mental health in the past month among US adults in 11 states and the District of Columbia. As a secondary aim, we assessed whether these associations differed by sex. We hypothesized that consuming any type of sugary beverage would be associated with greater prevalence of poor mental health, with larger magnitudes for women and for those who consume SSBs.

## Methods

### Data source and sample

We analyzed cross-sectional data from the Centers for Disease Control and Prevention’s (CDC’s) Behavioral Risk Factor Surveillance System (BRFSS). BRFSS is a validated cellular and landline telephone survey that collects information on health-related behaviors, chronic health conditions, and use of preventive services from more than 400,000 noninstitutionalized US adults each year. BRFSS uses disproportionate stratified sampling to collect a representative sample of the US population from all 50 states, the District of Columbia, and 3 US territories (21). The questionnaires include a core component, optional modules, and state-added questions. We used data from the District of Columbia and 11 states that included the optional SSB module in 2017: Alaska, Arizona, Delaware, District of Columbia, Hawaii, Iowa, New York, North Carolina, Ohio, Vermont, West Virginia, and Wisconsin. We included participants who responded to questions about poor mental health days and 100% fruit juice consumption on the core module and 2 SSB questions on the optional module (N = 68,819). We excluded those who responded “don’t know” or “not sure” to the mental health question (n = 688), the fruit juice question (n = 918), or either of the 2 SSB questions (n = 624), for a final study population of 66,589 respondents. This study was considered nonhuman subjects research by the University of Washington institutional review board because it is a secondary analysis of de-identified preexisting data to which none of the study personnel have access to identifiers.

### Exposure and outcome

Our exposures of interest were self-reported consumption of SSBs and 100% fruit juice. SSB consumption was pooled from 2 variables in the SSB optional module (“During the past 30 days, how often did you drink regular soda or pop that contains sugar?”; “During the past 30 days, how often did you drink sugar-sweetened fruit drinks (such as Kool-Aid and lemonade), sweet tea, and sports or energy drinks?”). Consumption of 100% fruit juice was based on the question, “Not including fruit-flavored drinks or fruit juices with added sugar, how often did you drink 100% fruit juice such as apple or orange juice?” Frequencies of SSB and 100% fruit juice responses were converted to daily beverage intake and categorized as 0 times per day, >0 to <1 times per day, or ≥1 times per day.

The outcome of this study was poor mental health, based on responses to the question, “Thinking about your mental health, which includes stress, depression, and problems with emotions, for how many days during the past 30 days was your mental health not good?” Mental health was dichotomized as fewer than 14 days or 14 or more days of poor mental health in the past month. We chose a cut-off point of 14 days on the basis of previous literature that suggests experiencing 2 or more weeks of poor mental health per month indicates significant mental distress, anxiety, or depression (22).

### Covariates

The confounders for our primary model were chosen a priori and included sex (male, female); age (18–24, 25–34, 35–44, 45–54, 55–64, ≥65 y); race/ethnicity (non-Hispanic White, non-Hispanic Black, non-Hispanic Asian, Hispanic, non-Hispanic American Indian/Alaska Native, non-Hispanic, other); education (less than a high school degree, high school degree, some college, college degree or more); employment (employed, unemployed, not in labor force [ie, defined as those who were unemployed and not seeking work]); smoking status (current, former, never); and physical activity (<150 min/week, ≥150 min/wk).

### Statistical analyses

To account for differences in nonresponse and selection probability, all statistical analyses included sample weights. Clustered-robust standard errors were implemented to account for survey clustering and homoskedasticity violations by using sampling strata and primary sampling units. We calculated the weighted prevalence of SSB and 100% fruit juice consumption overall and stratified by sociodemographic variables of interest. Sociodemographic differences across SSB and 100% fruit juice categories were assessed by examining 95% CIs produced from χ^2^ distributions. Differences in sociodemographic factors of greater than 5 percentage points across consumption categories were considered substantial. 

For our primary analysis, we used separate multivariable Poisson regression models with clustered-robust standard errors for SSB and 100% fruit juice to estimate adjusted prevalence ratios (PRs) and 95% CIs of poor mental health associated with each level of beverage consumption (0 vs <1 times per day and 0 vs ≥1 times per day). PRs were preferred to odds ratios because of the high prevalence of poor mental health in several categories. Poor mental health was treated as a binary outcome variable while SSB consumption and 100% fruit juice consumption were treated as indicator variables in their respective models. 

To examine potential effect modification of sex and SSB consumption, we used ANOVA with an F test to compare a model with interaction terms for sex with each exposure level of SSB to a nested model with no interaction terms. We used an analogous method to assess interaction in the 100% fruit juice analysis. We also conducted a sensitivity analysis that used higher thresholds for the 100% fruit juice consumption categories (<1, 1 to <2, 2 to <3, and ≥3 times per day) to examine juice in frequencies likely to exceed dietary recommendations because 100% juice is still considered healthy in small amounts. Similar to the primary analyses, we used multivariable Poisson regression to compare each frequency of 100% fruit juice consumption to the lowest category, less than 1 time per day. An ANOVA with an F test was used to compare a nested model to a model with an interaction term for sex and each exposure level of 100% fruit juice, by using a new 100% fruit juice variable with the 4 defined categories. Stratified estimates of the sensitivity analysis were explored to further examine sex-specific estimates of consuming higher thresholds of 100% fruit juice and poor mental health. All analyses were conducted in RStudio version 3.6.1 (RStudio, PBC) and significance was set at an α level of .05.

## Results

The weighted prevalence estimates of consuming no SSBs per day, consuming SSBs >0 to <1 times per day, and consuming SSBs ≥1 times per day were 29.1%, 44.9%, and 26.0%, respectively. In the fruit juice analysis, 38.3% of participants consumed no 100% fruit juice per day, 43.9% consumed 100% fruit juice >0 to <1 times per day, and 17.8% consumed 100% fruit juice ≥1 times per day (Table 1). A substantially higher prevalence of consuming SSBs ≥1 times per day versus none occurred among those who were aged 18 to 44, male, Black, Hispanic, high school graduates or less, current smokers, employed, and less physically active. Compared with those who did not consume 100% fruit juice, more people who consumed 100% fruit juice ≥1 times per day were male, Black, and more physically active (Table 1).

**Table 1:** Weighted Prevalence of Demographic, Behavioral, and Health Characteristics of US Adults, by Consumption Frequency of SSBs and 100% Fruit Juice, Behavioral Risk Factor Surveillance System, 2017

Characteristic^a^	Total No. (%)^b^	SSB Intake (times/d)^c^, % (95% CI)			100% Fruit Juice Intake (times/d)^d^, % (95% CI)		
		None (n = 23,474)	>0 to <1 (n = 28,165)	≥1 (n = 14,950)	None (n = 28,303)	>0 to <1 (n = 26,408)	≥1 (n = 11,878)
**Total sample**	66,589 (100.0)	29.1 (28.4–29.7)	44.9 (44.2–45.7)	26.0 (25.3–26.7)	38.3 (37.5–39.0)	43.9 (43.1–44.7)	17.8 (17.2–18.5)
**Age, y**							
18–24	3,243 (11.8)	4.9 (4.0–5.8)	14.3 (13.3–15.4)	15.2 (13.9–16.5)	8.9 (7.9–9.8)	14.4 (13.3–15.4)	11.7 (10.1–13.3)
25–34	6,115 (16.2)	8.8 (7.9–9.8)	18.7 (18.0–19.7)	20.3 (19.0–21.6)	12.8 (11.9–13.8)	19.5 (18.4–20.5)	15.6 (14.0–17.2)
35–44	7,501 (15.6)	11.8 (10.9–12.8)	16.2 (15.3–17.1)	18.5 (17.3–19.7)	14.6 (13.8–15.5)	17.3 (16.3–18.2)	13.1 (11.7–14.6)
45–54	10,578 (16.9)	17.5 (16.5–18.5)	16.2 (15.4–17.0)	17.4 (16.4–18.6)	17.7 (16.7–18.5)	17.2 (16.4–18.2)	14.6 (13.2–15.8)
55–64	14,760 (17.7)	23.8 (22.7–24.9)	16.3 (15.6–17.1)	13.4 (12.6–14.3)	21.4 (20.5–22.3)	15.1 (14.4–15.8)	16.4 (15.2–17.7)
≥65	24,392 (21.8)	33.1 (32.0–34.3)	18.3 (17.5–19.0)	15.1 (14.2–16.0)	24.6 (23.7–25.5)	16.5 (15.8–17.2)	28.6 (27.1–30.1)
**Sex**							
Male	29,056 (48.2)	38.8 (37.4–40.1)	49.7 (48.5–50.9)	56.0 (54.6–57.5)	41.4 (40.2–42.5)	52.2 (51.0–53.4)	52.9 (51.0–54.8)
Female	37,494 (51.8)	61.2 (60.0–62.6)	50.3 (49.1–51.5)	44.0 (42.5–45.5)	58.6 (57.5–59.8)	47.8 (46.7–49.0)	47.1 (45.2–49.0)
**Race/ethnicity**							
Non-Hispanic White	49,433 (70.2)	77.6 (76.3–78.9)	68.1 (66.9–69.2)	65.7 (64.2–67.2)	74.6 (73.5–75.8)	68.9 (67.8–70.1)	64.0 (62.1–65.9)
Non-Hispanic Black	5,385 (11.4)	7.7 (6.8–8.6)	11.8 (10.9–12.6)	14.9 (13.7–16.2)	8.3 (7.5–9.1)	12.4 (11.5–13.3)	15.6 (14.0–17.3)
Non-Hispanic Asian	2,846 (4.3)	4.2 (3.5–4.9)	5.3 (4.7–6.0)	2.5 (1.9–3.1)	4.8 (4.1–5.4)	4.1 (3.5–4.7)	3.5 (2.6–4.4)
Non-Hispanic AI/AN	1,387 (1.4)	1.0 (0.7–1.5)	1.3 (1.1–1.6)	2.1 (1.7–2.5)	1.4 (1.1–1.7)	1.5 (1.2–1.7)	1.5 (1.1–2.0)
Hispanic	4,303 (10.2)	7.5 (6.7–8.3)	11.0 (10.3–11.8)	11.7 (10.7–12.6)	8.8 (8.0–9.5)	10.4 (9.6–11.1)	12.8 (11.4–14.1)
Other	3,235 (2.5)	2.0 (1.6–2.3)	2.5 (2.2–2.8)	3.1 (2.6–3.7)	2.2 (1.9–2.5)	2.7 (2.4–3.1)	2.6 (2.0–3.2)
**Completed level of education**							
<High school diploma	4,222 (11.9)	9.5 (8.5–10.5)	10.1 (9.2–11.0)	17.8 (16.5–19.1)	11.2 (10.3–12.1)	11.0 (10.1–11.9)	15.7 (14.0–17.5)
High school diploma	18,268 (28.9)	25.0 (23.8–26.2)	26.5 (25.5–27.6)	37.4 (36.0–38.9)	29.0 (27.8–30.1)	28.2 (27.2–29.3)	30.6 (28.8–32.3)
Some college	18,358 (31.3)	30.0 (28.8–31.3)	32.7 (31.6–33.9)	30.1 (28.7–31.5)	29.8 (28.7–31.0)	33.1 (31.9–34.2)	29.8 (28.1–31.7)
≥College degree	25,553 (27.9)	35.5 (34.2–36.7)	30.7 (30.0–31.7)	14.7 (13.8–15.6)	30.0 (29.0–21.1)	27.7 (26.7–28.7)	23.9 (22.5–25.4)
**Smoking status**							
Current	9,838 (17.0)	10.7 (9.9–11.5)	13.5 (12.7–14.4)	30.1 (28.7–31.6)	15.6 (14.8–16.5)	18.1 (17.2–19.1)	17.2 (15.8–18.8)
Former	19,543 (25.4)	31.0 (29.8–32.3)	23.8 (22.8–24.7)	22.0 (20.8–23.2)	28.7 (27.6–29.7)	23.0 (22.0–23.9)	24.5 (22.9–26.1)
Never	36,886 (57.6)	58.3 (57.0–59.6)	62.7 (61.5–63.8)	47.9 (46.4–49.4)	55.7 (54.5–56.9)	58.9 (57.7–60.0)	58.3 (56.4–60.2)
**Employment^e^**							
Unemployed	2,941 (5.5)	4.4 (3.8–5.2)	4.9 (4.4–5.5)	7.7 (6.9–8.6)	4.9 (4.3–5.5)	5.9 (5.3–6.5)	5.9 (5.1–7.0)
Not in labor force	30,694 (37.7)	46.5 (45.2–47.9)	34.9 (33.7–36.0)	32.7 (31.3–34.2)	40.4 (39.2–41.6)	33.0 (31.9–34.1)	43.7 (41.8–45.6)
Employed	32,580 (56.8)	49.1 (47.7–50.4)	60.2 (59.0–61.4)	59.6 (58.1–61.0)	54.7 (53.5–56.0)	61.1 (60.0–62.3)	50.4 (48.5–52.4)
**Physical activity, min/wk**							
<150	29,850 (50.0)	44.7 (43.3–46.1)	49.0 (47.8–50.2)	56.5 (54.9–58.0)	50.5 (49.3–51.8)	50.6 (49.4–51.8)	45.6 (43.6–47.5)
≥150	36,649 (50.0)	55.3 (53.9–56.7)	51.0 (49.8–52.2)	43.5 (42.0–45.1)	49.5 (48.2–50.7)	49.4 (48.2–50.6)	54.4 (52.5–56.4)

Abbreviations: SSB, sugar-sweetened beverage; AI/AN, American Indian/Alaska Native.

a Number and percentage missing for other variables are as follows: age, n = 0 (0%); sex, n = 39 (0.1%); race/ethnicity, n = 0 (0%); education, n = 188 (0.3%); smoking, n = 322 (0.5%); physical activity, n = 2,090 (3.1%).

b All values for N are unweighted.

c SSBs include nondiet soda and fruit drinks that contain added sugar.

d Fruit juice includes drinks that contain 100% juice.

e “Not in labor force” denotes students, homemakers, retirees, and persons unable to work.

Poor mental health was prevalent in 10.2% of people who consumed no SSBs, 11.1% of those who consumed SSBs >0 to <1 time per day, and 17.5% of those who consumed SSBs ≥1 times per day (Table 2). Results from adjusted multivariable Poisson regression showed that people in the highest category of consumption (≥1 times per day) had a 26% higher prevalence of poor mental health compared with nonconsumers (PR = 1.26; 95% CI, 1.11–1.43). We did not observe any significant association between SSB intake of >0 to <1 times per day and poor mental health, compared with nonconsumers. The joint interaction term evaluating sex as a potential effect modifier of the relationship between SSB consumption and poor mental health was not significant (*P* = .89).

**Table 2:** Prevalence Ratios^a^ of Poor Mental Health Among US Adults, by Consumption Frequency of Sugar-Sweetened Beverages and 100% Fruit Juice, Behavioral Risk Factor Surveillance System, 2017

Variable	Weighted Prevalence of Poor Mental Health^b^, % (95% CI)^c^	Crude PR (95% CI)	Adjusted PR (95% CI)^d^	*P* Value
**SSB intake, times/d**				
None	10.2 (9.2–11.1)	1 [Reference]	1 [Reference]	.89^e^
>0 to <1	11.1 (10.3–11.9)	1.09 (0.97–1.22)	0.99 (0.88–1.11)	
≥1	17.5 (16.4 18.7)	1.72 (1.54–1.92)	1.26 (1.11–1.43)	
**100% fruit juice intake, times/d**				
None	12.9 (12.1–13.8)	1 [Reference]	1 [Reference]	.28^e^
>0 to <1	12.4 (11.6–13.2)	0.96 (0.88–1.05)	0.92 (0.83–1.00)	
≥1	11.8 (10.6–13.1)	0.91 (0.81–1.04)	0.89 (0.78–1.01)	

Abbreviations: PR, prevalence ratio; SSB, sugar-sweetened beverage.

a Regressions accounted for sampling weights. Clustered-robust standard errors were corrected for clustering.

b Poor mental health defined as 14 or more days in last 30 days that self-reported mental health was not good.

c Weighted prevalence estimates and associated 95% confidence intervals.

d Adjusted for age, sex, race/ethnicity, education, employment, smoking, and physical activity.

e Interaction by sex was assessed using a F test of joint significance, comparing a model with interaction terms for each intake category to a corresponding nested model without interaction terms.

For 100% fruit juice consumption, the weighted prevalence of poor mental health was 12.9% in nonconsumers, 12.4% in those who consumed >0 to <1 time per day, and 11.8% in those who consumed ≥1 times per day (Table 2). Results from the multivariable Poisson regression of 100% fruit juice and poor mental health indicated that frequency of 100% fruit juice consumption was not associated with poor mental health. Similar to the SSB analyses, the joint interaction term assessing multiplicative interaction between sex and 100% fruit juice consumption on the prevalence of poor mental health was not significant (*P* = .28).

Results from the sensitivity analysis examining higher frequencies of 100% fruit juice consumption and poor mental health suggested no association between any frequency of 100% fruit juice consumption and mental health (Table 3). For the examination of effect modification, the joint interaction term for sex and 100% fruit juice consumption was not significant (*P* = .08). In sex-stratified analyses, the prevalence of poor mental health among male and female respondents who consumed 100% fruit juice ≥3 times per day was 7.2% and 28.2%, respectively. No association between 100% fruit juice consumption and poor mental health was observed in male respondents (adjusted PR = 0.59; 95% CI, 0.30–1.16). Female respondents who consumed 100% fruit juice ≥3 times per day had a 52% higher prevalence of poor mental health compared with female respondents who consumed 100% fruit juice <1 time per day (adjusted PR = 1.52; 95% CI, 1.09–2.11) (Table 3). No association was found between lower categories of 100% fruit juice consumption (1 to <2, 2 to <3) and poor mental health in either of the sex-specific strata.

**Table 3:** Sensitivity Analysis Assessing Prevalence Ratios^a^ for Poor Mental Health Among US Adults, by Frequency of 100% Fruit Juice Consumption, Behavioral Risk Factor Surveillance System, 2017

Variable	Weighted Prevalence of Poor Mental Health^b^, % (95% CI)^c^	Crude PR (95% CI)	Adjusted PR (95% CI)^d^
**100% Fruit juice intake, times/d^e^**			
<1	12.7 (12.1–13.2)	1 [Reference]	1 [Reference]
1 to <2	11.3 (9.9–12.7)	0.89 (0.78–1.02)	0.94 (0.83–1.07)
2 to <3	12.4 (8.8–15.9)	0.98 (0.73–1.30)	0.86 (0.62 1.18)
≥3	16.6 (11.3–21.8)	1.31 (0.95 1.80)	1.09 (0.80–1.47)
Men			
<1	10.7 (9.9–11.5)	1 [Reference]	1 [Reference]
1 to <2	9.5 (7.7–11.3)	0.89 (0.72–1.08)	0.96 (0.79–1.17)
2 to <3	12.3 (7.3–17.2)	1.14 (0.76–1.72)	0.96 (0.61–1.52)
≥3	7.2 (2.8–11.7)	0.67 (0.36–1.25)	0.59 (0.30–1.16)
Women			
<1	14.4 (13.5 15.2)	1 [Reference]	1 [Reference]
1 to <2	13.2 (11.1–15.4)	0.92 (0.78–1.09)	0.93 (0.79–1.10)
2 to <3	12.5 (7.5–17.4)	0.87 (0.58–1.30)	0.74 (0.49–1.14)
≥3	28.2 (18.4–37.9)	1.96 (1.38–2.78)	1.52 (1.09–2.11)

Abbreviation: PR, prevalence ratio.

a Regressions accounted for sampling weights. Clustered-robust standard errors are corrected for clustering.

b Poor mental health defined as 14 or more days in last 30 days that self-reported mental health was not good.

c Weighted prevalence estimates and associated 95% confidence intervals.

d Adjusted for age, sex, race/ethnicity, education, employment, smoking, and physical activity.

e Interaction by sex was assessed using an F test of joint significance, comparing a model with interaction terms for each intake category to a corresponding nested model without interaction terms. *P* value for interaction by sex = .08.

## Discussion

We found that consuming SSBs ≥1 times per day versus none was associated with poor mental health whereas frequency of 100% fruit juice consumption was not associated with poor mental health. Our interaction analyses suggest that the associations between 100% fruit juice consumption and mental health did not differ significantly by sex.

Our findings linking daily SSB consumption to higher prevalence of poor mental health align with previous epidemiologic studies (9–11). We did not find any effect modification by sex, which is consistent with prior literature suggesting that the relationship between SSB consumption and mental health does not differ by sex (17,18). However, other research has found that women experience worse mental health associated with poor diet quality (23) and consumption of energy-dense foods (20). Prior research finding effect modification of diet and poor mental health by sex may be driven by broader dietary behaviors, such as caloric consumption and eating behavior, rather than consumption of particular foods such as sugary beverages.

To our knowledge, our study is the first to examine 100% fruit juice consumption and poor mental health. In our primary analyses, we did not observe an association between consuming 100% fruit juice ≥1 times per day and poor mental health, although this relationship may change for larger quantities of consumption. Because the SSB and 100% fruit juice questions only specified the “number of times per day” of consuming a given beverage, we did not have data on volume or sugar content. The average portion size of a soda is 20 ounces (24), much higher than a typical 8-ounce serving of juice. Consuming SSBs ≥1 times per day would likely equate to more sugar and calories than consuming 100% fruit juice ≥1 times per day.

Our finding that the association between consuming 100% fruit juice ≥3 times per day (vs <1 times per day) and poor mental health was significantly elevated when restricted to female but not male respondents may reflect differences in average body size. Men may require a higher frequency of sugary beverage consumption compared with women to elicit similar biological responses. Alternatively, females who consumed 100% fruit juice ≥3 times per day may experience enhanced feelings of guilt or stress in response to perceived overconsumption of 100% fruit juice. This explanation aligns with previous research suggesting that women experience worse guilt compared with men in response to high sugar consumption (17). These differential effects of perceived overconsumption in women versus men may not have been observable in the main analyses because the highest category for SSB and 100% fruit juice consumption was ≥1 times per day or possibly because both sexes may perceive SSBs as unhealthy whereas only women perceive 100% fruit juice as unhealthy. Because of differences in the direction of the associations by sex in stratified models, it may be of interest to examine sex-specific associations of consuming large quantities of sugary beverages and mental health, particularly in studies with larger sample sizes and more precise beverage consumption measurements.

Although we suspect that discrepancies in serving sizes may have obscured true comparisons between SSB and 100% fruit juice consumption and poor mental health, plausible reasons exist as to why consuming 100% fruit juice is associated with lower prevalence of poor mental health. Micronutrients, including vitamins in fruit juices, are important for brain function; several studies have linked deficiencies in vitamin B, a common nutrient in fruit juice, with depression (25,26). Another study found that patients with anxiety and depression had significantly lower levels of serum vitamin A, C, and E compared with healthy patients (27). Other researchers argue that different types of sugar elicit different physiological effects. Although 100% fruit juice contains a simple mixture of fructose, glucose, and sucrose, SSBs contain manufactured sweeteners such as high-fructose corn syrup, which may be difficult to metabolize (28). Fructose malabsorption has been linked to several risk factors for depression, including decreased tryptophan, an important precursor for mood-boosting serotonin (29).

Our study has limitations. First, the wording of the BRFSS questionnaire and reliance on self-reported data may have limited comparability of SSB and 100% fruit juice consumption. BRFSS asks only about frequency of SSB and 100% fruit juice consumption, which may have led to an underestimation of true SSB consumption compared with 100% fruit juice consumption. Furthermore, self-reported SSB consumption tends to be underreported (30). However, we found no literature to suggest that recall of SSB consumption differs by mental health status, indicating that no directional biases were introduced that would affect our results. Because of the lack of comparability across SSB and 100% fruit juice categories, we were unable to pool the exposures. Such an analysis would more completely assess the association between total sugary beverage intake and mental health. Second, the cross-sectional design precluded us from assessing temporality between sugary beverage consumption and mental health. Individuals with poor mental health may be more likely to choose beverages that are readily accessible and perceived as unhealthy; however, evidence exists for a temporal relationship between SSB consumption and mental health. One longitudinal study measuring SSB consumption and depression over several points found that SSB consumption preceded poor mental health (9). Despite these limitations, the large sample size, geographically diverse sample, and novel examination of 100% fruit juice present valuable additions to literature on sugary beverage consumption and mental health.

Our findings suggest that consuming SSBs 1 or more times daily is associated with poor mental health, while consuming 100% fruit juice with the same frequency is not. Although public health recommendations typically cite physical health consequences of consuming SSBs, practitioners should additionally consider the association between SSBs and mental health. Although our study may not have been sufficiently powered to include cut-off points for larger quantities of consumption in our primary model, our sensitivity analyses indicate that consumption of larger quantities of 100% fruit juice deserves closer examination. To improve understanding of the differential effect of SSBs and 100% fruit juice on mental health, future studies should leverage prospective designs to assess the relationship between consumption of specific volumes and subtypes of sugary beverages and poor mental health.
